# Supplementary material for: The influence of hip revision stem spline design on the torsional stability in the presence of major proximal bone defects
Source: PLoS One. 2023 Sep 14;18(9):e0291599. doi: 10.1371/journal.pone.0291599 (PMC10501563; doi:10.1371/journal.pone.0291599)
Supplement: Attachment — Submitted filename: Plosone2023August.docx [file pone.0291599.s002.docx]

Overall this is a great and useful article, which will enhance the knowledge of revision hip arthroplasty. Please find my comments below:

Line 54: This sentence about aseptic loosening should be rephrased, because as it is, it is incorrect.

Original: „Aseptic loosening is one of the major causes for hip re-revisions [3], which particularly occurs when adequate resistance to subsidence or torsional relative motion is not achieved postoperatively [9, 10]

Recommendation „ Aseptic loosening is one of the major causes for hip re-revisions [3], which usually occurs in the second or third decade following total hip replacement due to a biological process that weakens the bone-implant interface, mainly due to the immunological effect of wear particles (particle disease). Early aseptic loosening, is an avoidable complication, which occurs, when adequate resistance to subsidence or torsional relative motion is not achieved postoperatively[9, 10].

Line 62:

The following sentence might be confusing to orthopaedic surgeons as it is somewhat vague.

„The suggested length over which the implant should engage with the bone ranges from 10 mm to 80 mm [15, 16].”

The two articles quoted are 25 years apart and they talk about 2 different uncemented stem anchoring phiosophies.Yes, the scratch fit philosophy (cylindrical stems) requires long bone to implant contact, whilst with the taper fluted design it can be as little as 3 cms.

Recommendation:

The suggested length over which the implant should engage with the bone ranges from as little 20 mm for taper flutted designs, to as long as 80 mm for cylindrical uncemented femoral stems[15, 16].

Figure 2 a and 2 b are missing. These pictures are important for the reader to understand the difference better. How many splines are there for the prototype? 16? (Reclaim has 8).

Line 102-105

It is not clear how the reaming was performed. Was it hand reaming or power reaming, or perhaps both? Was the final position determined by feel for the cortical contact and establishing a femoral cone fit? Was the reaming performed with the same reamers for both stem designs? This is an important issue.

Line 244-246

The contact area, as measured by the number of matrix segments with contact between the stem and the cortical femoral canal, was 54 % greater for the Prototype stem compared to the Reclaim stem (p = 0.046, dependent t-test, Fig 9B).

Comment: the contact pattern as seen on Figure 6 shows two distinctly differenct „contact maps”, the prototype seems (Figure 6A) to have contact at the distal point of the ETO then, a large contact „patch” distally between 120-240 degrees. The Reclaim stem shows a more even distribution of contact points (Figure 6B) although the areas are smaller. How do the investigatiors explain this? Are there any other differences between the stem designs (shape, tip of the stem, bevel?)? Am I interpreting Figure 6 correctly?

Line 275

„In support of this hypothesis, despite higher implant forces for the prototype stems, no fractures occurred with either design”

Although this sentence might be techincally correct, it should be interpreted with caution as there were only 10 femurs, and in vivo use of the implant might still provide suprises.
